# *In vivo* Host Environment Alters *Pseudomonas aeruginosa* Susceptibility to Aminoglycoside Antibiotics

**DOI:** 10.3389/fcimb.2017.00083

**Published:** 2017-03-14

**Authors:** Xiaolei Pan, Yuanyuan Dong, Zheng Fan, Chang Liu, Bin Xia, Jing Shi, Fang Bai, Yongxin Jin, Zhihui Cheng, Shouguang Jin, Weihui Wu

**Affiliations:** ^1^State Key Laboratory of Medicinal Chemical Biology, Key Laboratory of Molecular Microbiology and Technology of the Ministry of Education, Department of Microbiology, College of Life Sciences, Nankai UniversityTianjin, China; ^2^State Key Laboratory of Medicinal Chemical Biology and College of Pharmacy and Life Sciences, Nankai UniversityTianjin, China; ^3^Department of Molecular Genetics and Microbiology, College of Medicine, University of FloridaGainesville, FL, USA

**Keywords:** infection, *Pseudomonas aeruginosa*, host cells, tobramycin, neutrophil, membrane potential

## Abstract

During host infection, *Pseudomonas aeruginosa* coordinately regulates the expression of numerous genes to adapt to the host environment while counteracting host clearance mechanisms. As infected patients take antibiotics, the invading bacteria encounter antibiotics in the host milieu. *P. aeruginosa* is highly resistant to antibiotics due to multiple chromosomally encoded resistant determinants. And numerous *in vitro* studies have demonstrated the regulatory mechanisms of antibiotic resistance related genes in response to antibiotics. However, it is not well-known how host environment affects bacterial response to antibiotics. In this study, we found that *P. aeruginosa* cells directly isolated from mice lungs displayed higher susceptibility to tobramycin than *in vitro* cultured bacteria. *In vitro* experiments demonstrated that incubation with A549 and differentiated HL60 (dHL60) cells sensitized *P. aeruginosa* to tobramycin. Further studies revealed that reactive oxygen species produced by the host cells contributed to the increased bacterial susceptibility. At the same concentration of tobramycin, presence of A549 and dHL60 cells resulted in higher expression of heat shock proteins, which are known inducible by tobramycin. Further analyses revealed decreased membrane potential upon incubation with the host cells and modification of lipopolysaccharide, which contributed to the increased susceptibility to tobramycin. Therefore, our results demonstrate that contact with host cells increased bacterial susceptibility to tobramycin.

## Introduction

*Pseudomonas aeruginosa* is an opportunistic bacterial pathogen which causes acute and chronic infections in human. It is one of the major pathogens causing nosocomial infections (Driscoll et al., 2007; De and Plésiat, 2011). *P. aeruginosa* possesses multiple antibiotic resistance mechanisms, such as low membrane permeability, antibiotic inactivating enzymes, multidrug efflux systems, and biofilm formation (Morita et al., 2014).

Genes involved in antibiotic resistance are tightly controlled by various regulatory pathways in response to antibiotic induced stresses (Poole, 2011). For example, β-lactam antibiotics inhibit peptidoglycan crosslink, leading to aberrant accumulation of muropeptides in cytoplasm, which activates AmpR, a LysR-type transcriptional regulator. Upon activation, it directly up regulates the expression of a chromosomally encoded β-lactamase AmpC, thus enhancing bacterial resistance to β-lactam antibiotics (Kong et al., 2005). Aminoglycoside antibiotics inhibit translation. Stalling of ribosome in the leader peptide of PA5471.1 activates the transcription of downstream gene (*PA5471*) through a transcription attenuation mechanism (Morita et al., 2009). The PA5471 protein binds to MexZ, releasing it from the promoter of *mexX*-*mexY* genes, which encode an efflux pump that is mainly responsible for bacterial resistance against aminoglycoside antibiotics (Morita et al., 2012a,b). In addition, it has been demonstrated that sub-inhibitory concentrations of tobramycin, ciprofloxacin, or tetracycline enhanced *P. aeruginosa* biofilm formation (Hoffman et al., 2005; Linares et al., 2006).

Similar to antibiotics, host environment also imposes stresses to the invading bacteria, such as antimicrobial peptides, low iron environment, reactive oxygen species (ROS) generated by phagocytes. In response, bacteria orchestrate the production of a variety of virulence factors to counteract host defense mechanisms (Lyczak et al., 2000). Upon contact with host cells, the type III secretion system (T3SS) of *P. aeruginosa* is activated, which injects effector proteins into host cells, causing alteration of cell signaling or cell death (Hauser, 2009). It has been demonstrated in a murine acute pneumonia model that *P. aeruginosa* preferentially injects T3SS effector proteins into neutrophils (Geddes et al., 2008; Berube et al., 2016). Expression of the T3SS is regulated by multiple regulatory pathways. Small RNAs RsmY and RsmZ reciprocally regulate T3SS and biofilm formation (Gooderham and Hancock, 2009). The alginate regulatory factor AlgU negatively regulates T3SS (Intile et al., 2014). And the cAMP receptor protein Vfr activates the expression of T3SS genes. In addition, Vfr is required for flagellum, pilus biosynthesis, type II secretion system, and the expression of exotoxin A. Exotoxin A is also under the regulation of PvdS (Ochsner and Vasil, 1996). In response to the host low iron environment, PvdS activates siderophore biosysnthesis for acquisition of iron, which is essential for the bacterial growth in host (Leoni et al., 2000; Wilson et al., 2001). The above examples suggest that bacterial virulence factors are regulated by a complicated regulatory network in response to the adverse host environment. However, how the regulatory network of virulence factors interweaves with that of the antibiotic resistance determinants remains to be studied.

In clinic settings, patients are usually prescribed with antibiotics after indications of bacterial infections, and as a result, the invading bacteria encounter antibiotics within the host milieu. We hypothesized that the global gene expression shaped by the host environment might influence bacterial resistance to antibiotics. Here in this study, we compared survival rates between *in vitro* grown bacteria and those isolated from mice and found that the host environment indeed affected bacterial resistance against antibiotics. To explore the mechanism, we compared gene expression profiles after treatment with tobramycin in the presence or absence of epithelial cell line A549 and differentiated HL60 cells (as neutrophils). Contact with host cells affected the expression of genes involved in LPS modification as well as the bacterial membrane potential. Our results shed light on how host environment affects bacterial susceptibility to antibiotics.

## Materials and methods

### Ethics statement

All animal studies complied with National and Nankai University guidelines regarding the use of animals in research. All animal experiments protocol was approved by the institutional animal care and use committee of the college of life sciences of Nankai University with the permit number NK-04-2012.

### Bacterial strains and plasmids

The bacterial strains used in this study are listed in Table S1. Bacteria were cultured in Luria–Bertani (LB) broth (10 g/l tryptone, 5 g/l yeast extract, 5 g/l Nacl, pH 7.0–7.5; Oxoid Ltd, USA) or LB agar (LB broth containing 15 g/l agar) under aerobic condition at 37°C. Antibiotics were used at the following concentrations: for *P. aeruginosa*, gentamicin at 100 μg/ml, carbenicillin at 150 μg/ml; for *E. coli*, ampicillin at 100 μg/ml. All antibiotics were purchased from BBI life sciences, Shanghai, China.

Plasmids used in this study are listed in Table S1. For DNA manipulation, standard protocols or manufacturer's instructions of commercial products were followed. A region containing the *waaP* gene and 50 bp upstream of the start codon was amplified by PCR and cloned into the *Sal*I, *Xba*I sites of pRKaraRed, resulting in pE2106, where the *waaP* gene is under the control of an arabinose-inducible promoter and fused with a His tag on the C terminus. An IPTG-inducible expression vector that expresses antisense RNA (asRNA) with paired termini (PT) against *waaP* was constructed as previously described (Nakashima and Tamura, 2009). The fragment of PT and a transcription terminator T0 (Nakashima and Tamura, 2009) was synthesized and cloned into the *EcoR*I-*Hind*III sites of pMMB67EH, resulting in pE1992. A fragment covering 35 bp upstream of the start codon and the first 65 bp of the *waaP* gene was PCR amplified and cloned into *Nco*I-*Xho*I sites inside of PT fragment of pE1992, resulting in pP2804.

### Cell culture and HL60 differentiation

HL60 and A549 cells were cultured in RPMI-1640 medium with 10% fetal bovine serum (FBS) at 37°C with 5% CO_2_, supplemented with penicillin G (100 U/ml) and streptomycin (100 μg/ml; Hyclone, USA). HL60 cell differentiation was performed as previously described (Sun et al., 2014).

### *In vitro* infection and bacteria susceptibility assay

A549 cells (1 × 10^4^) were seeded into each well of a 96-well plate, and incubated at 37°C with 5% CO_2_ for 16–20 h. The cells were washed twice with Hank's balanced salt solution (HBSS; Hyclone, USA) before bacterial infection. Overnight bacterial culture was sub-cultured in fresh LB broth and grown to an OD_600_ of 1.0. Bacteria were washed once and resuspended in HBSS to a concentration of 1 × 10^8^ CFU/ml. One hundred microliters bacterial suspension was added to A549 cells and incubated for 10 min, followed by addition of 1 × 10^6^ differentiated HL60 cells. Twenty minutes later, antibiotics at indicated concentrations were added to each well and incubated at 37°C. After 50 min, the number of live bacteria was determined by serial dilution and plating. The bacterial survival rate was calculated by dividing the number of live bacteria in the presence of antibiotic by that in the absence of antibiotic.

### Measurement of ROS level

ROS level was measured as previously described (Sun et al., 2014) with minor modification. A549 cells in each well of a 96-well plate were washed twice with warm HBSS. Then, 100 μl HBSS containing 100 μM luminol and 5 units of horseradish peroxidase (Sigma) was added to each well. For differentiated HL60 cells, 1 × 10^6^ cells in 100 μl of HBSS containing 100 μM luminol and 5 units of horseradish peroxidase were added to each well and incubated for 10 min at 37°C. PAO1 in HBSS (1 × 10^7^ CFU) were added into each well, and the ROS level was monitored every 2–5 min for 2 h with a luminometer (Varioskan Flash, Thermo Scientific).

### Reverse transcription and real time PCR

Total RNA was isolated using TRIzol (Thermo Fisher Scientific, USA) and a Direct-Zol RNA Miniprep (Zymo Research, USA). Total RNA from cells and bacteria mixtures were immediately treated with a MICROB *Enrich* (Life Technologies, USA) kit to remove RNA from eukaryotic cells. cDNA was synthesized with PrimeScript Reverse Transcriptase (TaKaRa, Dalian, China) and random primers. cDNA was mixed with 10 μl SYBR Premix Ex TaqTM II (TaKaRa) and 4 pmol forward and reverse primers (Table S1) in a total volume of 20 μl. The 30S ribosomal protein gene *rpsL* was used as an internal control in each real time PCR assay. Real time PCR was performed with a CFX Connect Real-Time system (Bio-Rad, USA).

### RNA sequencing and data analysis

Total RNA was isolated using the Trizol Reagent (Thermo Fisher Scientific, USA). The quantity and integrity were determined using a NanoDrop spectrophotometer (Thermo Scientific) and a Bioanalyzer 2100 system (Agilent). For mRNA sequencing, a Ribo-Zero rRNA Removal Kit (Illumina, San Diego, CA, USA) was used to selectively remove rRNA. Random oligonucleotides and SuperScript III were used to synthesize the first strand cDNA. Second strand cDNA synthesis was subsequently performed using DNA Polymerase I and RNase H. Remaining overhangs were converted into blunt ends via exonuclease/polymerase treatment. After adenylation of the 3′ ends of the DNA fragments, Illumina PE adapter oligonucleotides were ligated to prepare for hybridization. To select cDNA fragments around 300 bp in length, the library fragments were purified using the AMPure XP system (Beckman Coulter, Beverly, CA, USA). DNA fragments with ligated adaptor molecules on both ends were selectively enriched using Illumina PCR Primer Cocktail in a 15 cycle PCR reaction. The products were purified with the AMPure XP system and quantified using the Agilent high sensitivity DNA assay on a Bioanalyzer 2100 system (Agilent). The DNA library was then sequenced on a NextSeq 500 platform (Illumina) by Shanghai Personal Biotechnology Cp. Ltd.

Sequence reads were mapped onto PAO1 reference genome (NC_002516.2) using a CLC genomics Workbench 8.0 (CLC Bio-Qiagen, Aarhus, Denmark). The count data of expression values were then analyzed using a DESeq package of R/Bioconductor. The differentially expressed genes were identified by performing a negative binomial test using the DESeq software, with the cut-off fold-change larger than 2. The raw sequence reads were normalized by dividing with size factors, then Log2 (N + 1) transformed. The data have been deposited in the NCBI Short Read Archive (SRA) database with an accession number SRP100146.

### Membrane potential assay

Bacteria with or without incubation with eukaryotic cells were diluted to 10^6^ CFU/ml in HBSS. One microliter 20 mM DiOC2(3) was incubated with the bacteria in a 1 ml volume and incubated for 20 min at room temperature in dark, followed by flow cytometry analyses (Accuri C6, BD). DiOC2(3) was excited at 484 nm and emissions at 530 nm (Green fluorescence) and 610 nm (Red fluorescence) were measured. Membrane potential was indicated by ratios of Green and Red fluorescence (Novo et al., 1999).

### Down regulation of *waaP* by antisense RNA

Antisense RNA (AsRNA) construction was performed as previously described (Nakashima and Tamura, 2009). A fragment containing 35 bp upstream of the start codon and the first 65 bp of the *waaP* gene was amplified using PAO1 genomic DNA as the template. The fragment was cloned into pE1992, in which an RNA complementary to the *waaP* mRNA is driven by an inducible *tac* promoter. The construct was confirmed by DNA sequencing. To examine the inhibitory effect of the AsRNA, the *waaP* gene including its upstream 50 bp was fused with a 6 × HIS tag at the 3′ terminus (*waaP*-His) and cloned into pRKaraRed (Liang and Liu, 2010; Verstraeten et al., 2015), where expression of the gene is driven by an arabinose inducible promoter (P_BAD_). The two plasmids were co-transformed into PAO1, and the levels of WaaP-His was determined by Western blot.

### Western blotting

*P. aeruginosa* strains were grown in LB overnight at 37°C and then diluted 50-fold in fresh LB with 1 mM IPTG and 0.2% (vol/vol) arabinose, and cultured for 3 h. Samples from equivalent numbers of bacterial cells were separated by sodium dodecyl sulfate polyacrylamide gel electrophoresis (SDS-PAGE). The proteins were transferred onto a polyvinylidene difluoride (PVDF) membrane (Millipore, USA) and probed with a rabbit polyclonal antibody against 6 × HIS (Cell Signaling Technology, USA). The luminescence signals were detected with an ECL-plus kit (Millipore, USA).

### *In vivo* infection

Mouse acute pneumonia was performed as previously described (Sun et al., 2014). *P. aeruginosa* strains were grown in LB overnight and then subcultured into fresh medium and grown at 37°C with aeration to an OD_600_ of 1.0. Bacteria were washed and adjusted to 2 × 10^9^ CFU/ml in phosphate-buffered saline (PBS). Each female BALB/c mouse (6–8 weeks old) was anesthetized with an intraperitoneal injection of 5 μl/g 7.5% chloral hydrate. Then 20 μl bacterial suspension was intranasally inoculated into each anesthetized mouse, 10 μl per nostril. Bronchi alveolar lavage fluid (BALF) was collected as previously described (Wu et al., 2012). Briefly, 6 h post infection, mice were sacrificed by inhalation of CO_2_. Two microliters PBS containing 0.05 mM EDTA was injected into lungs through trachea by a vein detained needle (BD, Angiocath). After 1 min detaining, fluid was collected.

## Results

### Tobramycin susceptibility of bacteria isolated from BALF

**Figure 1:**
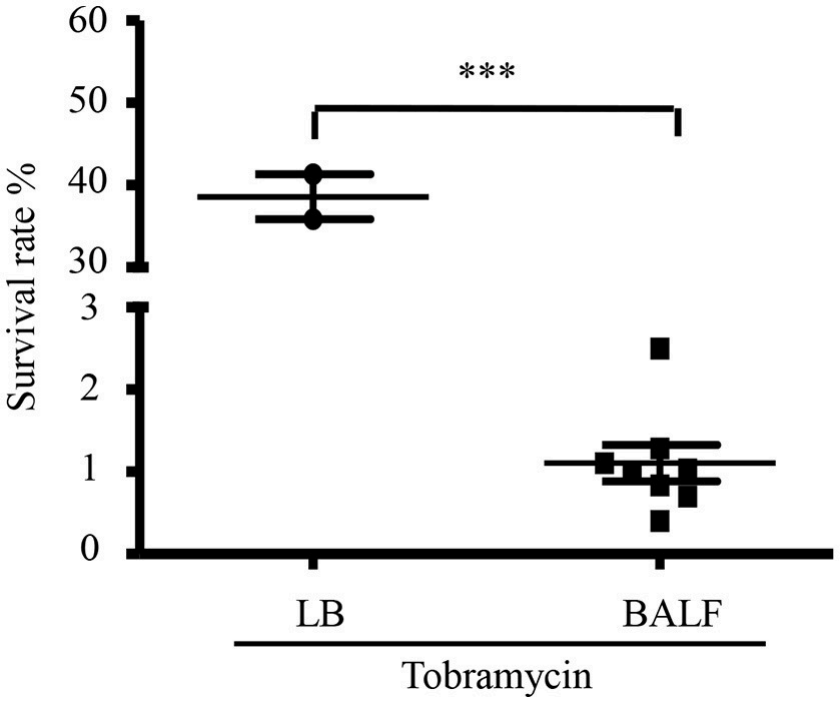
**Tobramycin susceptibility of bacteria isolated from BALF**. Survival rate of PAO1 grown in LB or isolated from BALF. Mice were infected with 4 × 10^7^ PAO1 intranasally. 6 h post infection, bacteria were isolated from BALF, resuspended in HBSS and treated with 2 μg/ml tobramycin for 50 min. Bacteria grown in LB were resuspended in HBSS and treated with 2 μg/ml tobramycin for 50 min. Number of live bacteria was determined by serial dilution and plating. Error bars represent the standard deviation. ^***^P < 0.001 by Student's *t*-test.

To examine whether host environment affects *P. aeruginosa* susceptibility to antibiotics, we infected mice with a wild type *P. aeruginosa* strain PAO1 itranasally and isolated bacteria from BALF 6 h post infection. The bacteria were treated with tobramycin, which is used in clinic to treat patients infected by *P. aeruginosa*. As a comparison, similar numbers of bacteria grown in LB were treated with tobramycin. The number of bacteria in each sample was listed in Table S2. Interestingly, the isolated bacteria showed higher susceptibility to tobramycin than those grown *in vitro* (Figure 1), suggesting that host environment might affect bacterial susceptibility to antibiotics.

### Interaction with differentiated HL60 cells increased bacterial susceptibility to tobramycin

**Figure 2:**
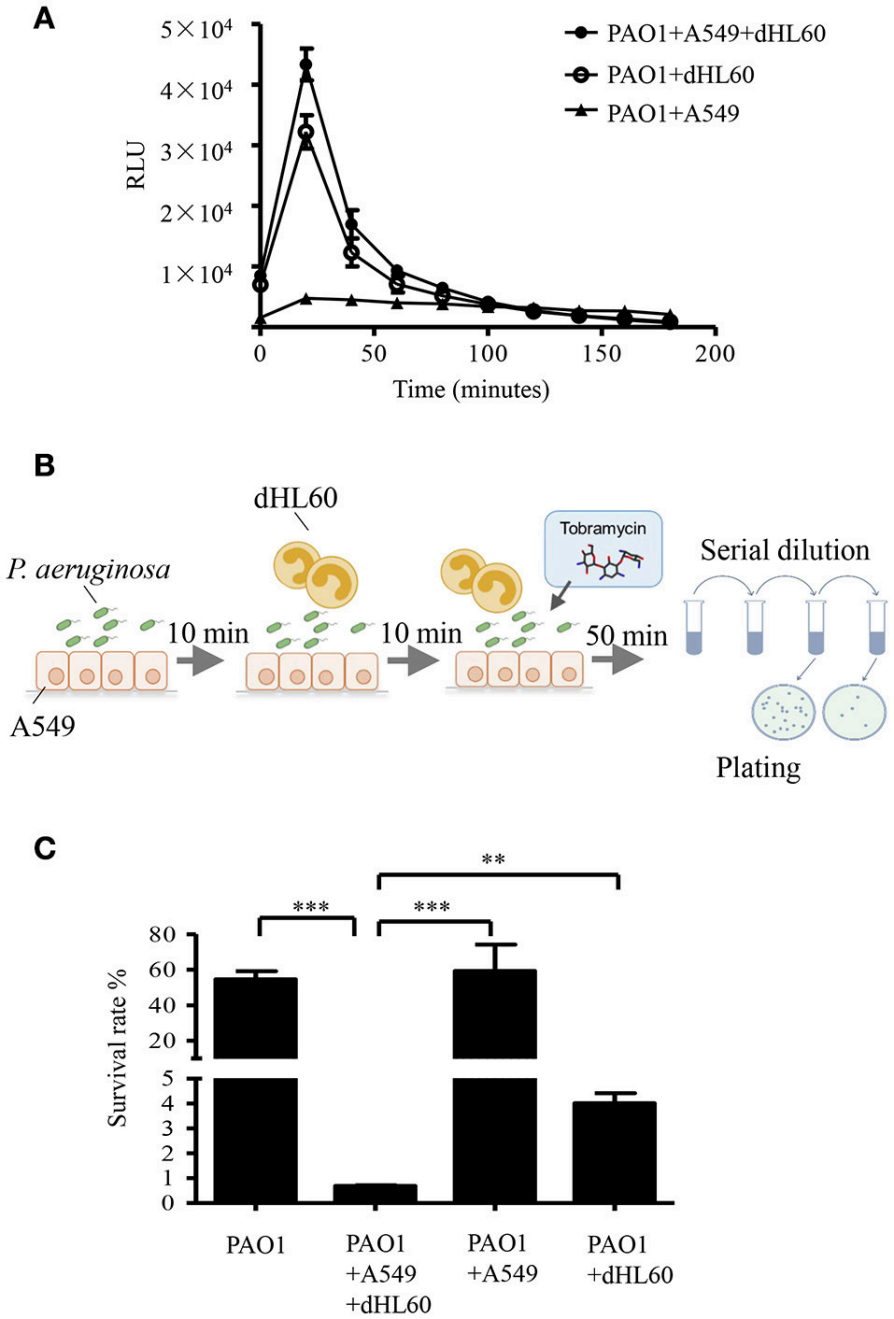
**Interaction with host cells increases bacterial susceptibility to tobramycin. (A)** ROS production by A549, dHL-60 or dHL-60 with A549 cells with or without PAO1 infection (MOI = 10). RLU, relative light units. **(B)** Schematic diagram of *in vitro* cell infection and antibiotics treatment assay on *P. aeruginosa*. PAO1 cells (1 × 10^7^) were incubated with A549 (4 × 10^4^) for 10 min, followed by addition of 1 × 10^6^ dHL60 cells. After 10 min, 2 μg/ml tobramycin was added. Then the bacteria and cells were incubated for another 50 min. The number of live bacteria was determined by serial dilution and plating. **(C)** PAO1 alone or PAO1 incubated with indicated cells and treated with 2 μg/ml tobramycin as illustrated in **(B)**. Same number of bacteria were used in the survival assay with our without the host cells. The bacterial survival rate was calculated by dividing the number of live bacteria in the presence of tobramycin by that incubated with same eukaryotic cells in the absence of tobramycin. Error bars represent the standard deviation. ^***^P < 0.001; ^**^P < 0.01 by Student's *t*-test.

To explore the mechanism of the increased bacterial susceptibility, we treated wild type PAO1 with tobramycin in the presence or absence of mammalian cells *in vitro*. Previously in a murine acute pneumonia model, it was demonstrated that neutrophils are recruited to the lung after infection with *P. aeruginosa* (Diaz and Hauser, 2010; Sun et al., 2014). These results indicate that the invading bacteria contact with epithelial cells first, followed by encountering neutrophils which generate reactive oxygen species (ROS) and release proteases and bactericidal peptides locally (Weiss, 1989). To mimic the *in vivo* infection scenario, we infected the human alveolar adenocarcinoma (A549) cells with PAO1 for 10 min, and then added neutrophil-like differentiated HL60 cells (designated as dHL60 hereafter). The response of dHL60 cells to *P. aeruginosa* was monitored by measuring ROS production. As shown in Figure 2A, dHL60 cells generated significantly higher amount of ROS than A549 cells upon encountering *P. aeruginosa*. Meanwhile, live bacteria number was determined 50 min after the addition of dHL60 cells. As shown in Figure S1, presence of A549 and dHL60 cells did not affect bacterial survival (Figure S1).

We next treated PAO1 with 2 μg/ml tobramycin for 10 min followed by the addition of dHL60 cells, when the ROS production reached the peak (Figure 2A). Fifty minutes later, the bacterial survival rates were determined (Figure 2B). As a comparison, similar numbers of bacteria grown in LB were treated with tobramycin for the same period of time. Compared to bacteria alone, incubation with A549 and dHL60 cells reduced the bacterial survival rate by ~80-fold (Figure 2C).

To dissect the role of each type of cell in the increased bacterial susceptibility, PAO1 were incubated with A549 or dHL60 cells individually, followed by treatment with tobramycin. A549 cells did not affect the bacteria survival rate, whereas dHL60 cells reduced the survival rate by ~20-fold (Figure 2C). These results suggest that dHL60 cells play a major role in the increased bacterial susceptibility to tobramycin, and the presence of A549 cells further increased the bacterial susceptibility.

### ROS contributes to increased bacterial susceptibility to tobramycin

**Figure 3:**
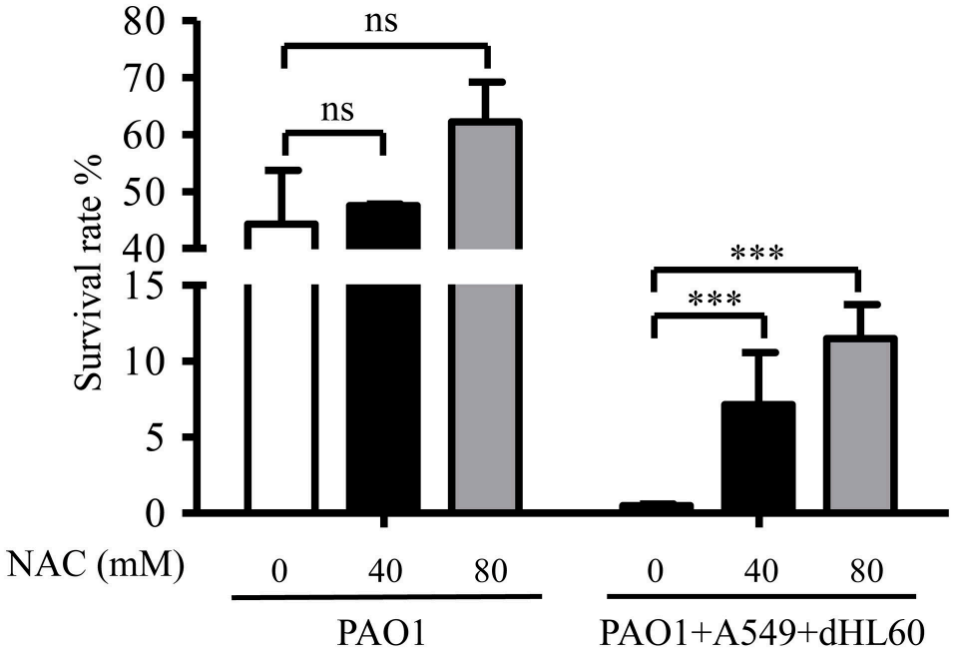
**ROS contributes to increased bacterial susceptibility to tobramycin**. PAO1 was incubated with A549 and dHL60 cells and treated with tobramycin as described in Figure 1. When dHL60 cells were added, 6 N-Acetyl-L-cysteine (NAC) at indicated concentration was also added to the mixture. The bacterial survival rate was calculated by dividing the number of live bacteria in the presence of tobramycin by that incubated with same eukaryotic cells in the absence of tobramycin. Same number of bacteria were used in the survival assay with our without the host cells. Error bars represent the standard deviation. ^***^P < 0.001 by Student's *t*-test.

As shown in Figure 2, dHL60 cells generate large amount of ROS upon encountering bacteria. To examine whether ROS plays a role in sensitizing bacteria to tobramycin, we used 6 N-Acetyl-L-cysteine (NAC) to neutralize ROS (Curtin et al., 2002). NAC alone did not alter bacterial survival rate in the absence of dHL60 cells, however, in the presence of dHL60 cells, NAC increased the survival rate in a dose dependent manner (Figure 3). To confirm the role of ROS, we treated bacteria with various concentrations of H_2_O_2_ in the presence or absence of tobramycin. At the concentration of 0.25 mM, H_2_O_2_ alone did not affect bacterial survival, but reduced the survival rate in the presence of tobramycin (Figure S2). These results suggest that ROS contributes to the increased bacterial susceptibility to tobramycin.

### Transcriptome analysis of bacteria incubated with mammalian cell and/or tobramycin

To further explore the mechanism of the increased bacterial susceptibility to tobramycin, we compared the transcription profiles among the following samples: PAO1, PAO1+tobramycin, PAO1+A549+dHL60, PAO1+A549+dHL60+tobramycin. In a previous transcriptome analysis of PAO1 in response to tobramycin, bacteria were treated with 2 μg/ml tobramycin for 30 min in BBL cation-adjusted Mueller-Hinton broth medium (CAMHB) before RNA isolation (Kindrachuk et al., 2011). We tested the survival rates of PAO1 after treatment with 2 μg/ml tobramycin for various periods of time in HBSS. After 20-min treatment, the bacterial survival rate was ~80% (Figure S3A) and we chose this time point for RNA isolation. Next, we determined the bacterial survival rates in the presence of A549 and dHL60 cells after treatment with various concentrations of tobramycin for 20 min. As shown in Figure S3B, treatment with 0.3 μg/ml tobramycin resulted in 80% live bacteria. Thus, this condition was chosen for RNA isolation. To compare the bacterial responses to the same concentration of tobramycin with or without A549 and dHL60 cells, we included a bacterial sample treated with 0.3 μg/ml tobramycin for 20 min in HBSS. The experimental conditions for RNA sample collection are summarized in Table 1.

**Table 1:** **Expression of heat shock, molecular chaperone, LPS O-antigen biosynthesis, and LPS phosphorylation genes in response to Tobramycin and host cells**.

**Gene ID**	**Name**	**Description**	**Sample Name**							
			**P0.3**		**P2**		**APD0**		**APD0.3**	
			**Components**							
			**PAO1**		**PAO1**		**PAO1**		**PAO1**	
			**0.3 μg/ml Tobramycin**		**2 μg/ml Tobramycin**		**No Tobramycin A549 and dHL60**		**0.3 μg/ml Tobramycin A549 and dHL60**	
**Tobramycin response**			Fold change^*^	***P*-value**	Fold change^*^	***P*-value**	Fold change^*^	***P*-value**	Fold change^*^	***P*-value**
PA3126	*ibpA*	heat shock protein IbpA	0.94	6.40E-01	1.44	9.69E-03	0.81	9.80E-01	2.05	6.42E-14
PA4385	*groEL*	molecular chaperone GroEL	1.12	2.70E-01	2.12	1.78E-19	1.43	5.27E-10	4.11	3.33E-122
PA4386	*groES*	co-chaperonin GroES	1.20	6.82E-02	2.55	6.71E-30	1.40	1.05E-08	3.88	1.48E-103
PA4760	*dnaJ*	molecular chaperone DnaJ	1.21	4.61E-01	2.49	1.77E-05	3.89	8.0380E-17	8.22	1.01E-50
PA4761	*dnaK*	molecular chaperone DnaK	1.12	4.35E-01	2.19	3.31E-11	1.16	9.73E-03	4.13	4.79E-61
PA4762	*grpE*	heat shock protein GrpE	1.33	2.22E-01	3.19	8.51E-10	2.32	2.2053E-07	6.93	7.25E-45
PA5053	*hslV*	ATP-dependent protease peptidase subunit	1.04	9.25E-01	2.71	8.79E-05	5.16	5.07E-18	11.90	6.68E-55
PA5054	*hslU*	ATP-dependent protease ATP-binding subunit HslU	1.18	6.38E-01	2.95	9.95E-05	4.95	6.92E-14	11.30	1.22E-41
**LPS O-ANTIGEN BIOSYNTHESIS**										
PA3149	*wbpH*	glycosyltransferase WbpH	0.56	1.48E-02	0.16	2.37E-08	2.81	1.00E-14	2.98	8.4451E-17
PA3150	*wbpG*	LPS biosynthesis protein WbpG	0.91	5.38E-01	0.15	2.46E-15	3.02	2.56E-30	2.84	9.6281E-28
PA3153	*Wzx*	O-antigen translocase	0.96	8.49E-01	0.01	7.79E-09	2.13	5.49E-06	2.18	1.76E-06
PA3154	*Wzy*	B-band O-antigen polymerase	1.24	4.39E-01	0.12	1.89E-05	3.89	4.96E-14	4.51	5.1553E-18
**LPS PHOSPHORYLATION**										
PA5006		hypothetical protein	0.90	6.65E-01	1.01	9.93E-01	4.34	3.3053E-24	4.79	7.7100E-29
PA5007	*wapG*	hypothetical protein	0.95	8.34E-01	0.81	4.72E-01	4.01	2.0919E-15	5.14	6.2265E-23
PA5008	*wapP*	hypothetical protein	1.08	7.17E-01	0.83	3.52E-01	1.69	3.22E-05	2.08	9.1969E-09
PA5009	*waaP*	lipopolysaccharide kinase WaaP	1.15	7.29E-01	0.76	5.12E-01	7.02	1.5556E-18	5.98	3.2275E-15

* *The fold change was calculated by dividing the mRNA level in each sample by the value of PAO1 incubated in HBSS alone (sample P0 in Table S3). The mRNA levels of each sample were shown in Table S3*.

**Figure 4:**
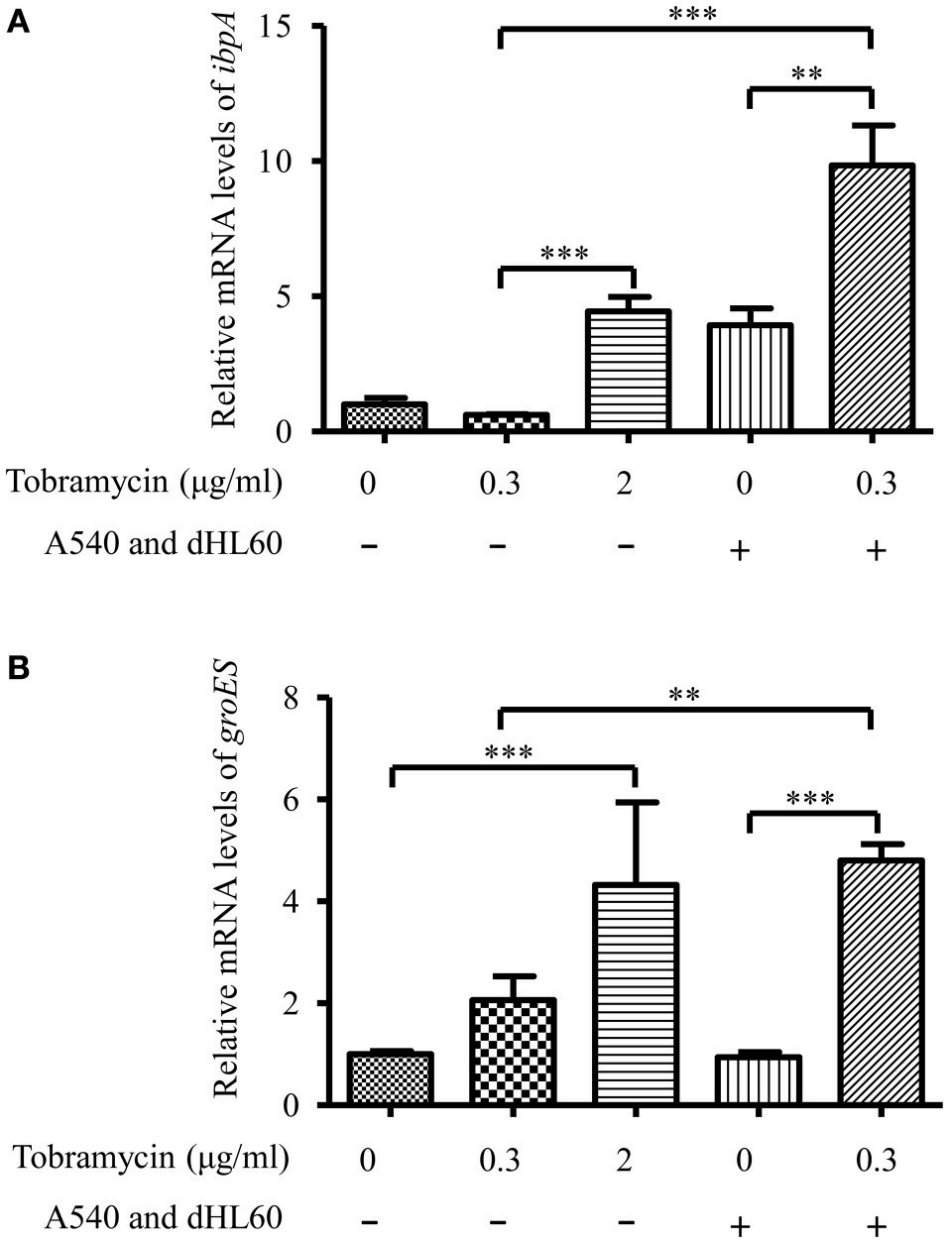
**mRNA levels of *ibpA* and *groES* during *in vitro* cell infection**. PAO1 was treated with 0.3 μg/ml of tobramycin for 20 min, followed by RNA isolation. For treatment with tobramycin in the presence of host cells, PAO1 (1 × 10^7^) was incubated with 4 × 10^4^ A549 cells for 10 min, followed by addition of 1 × 10^6^ dHL60 cells. After 20 min, 0.3 μg/ml of tobramycin was added to the mixture and incubated for 20 min, followed by RNA isolation. The mRNA levels of *ibpA* and *groES* were determined by real-time PCR. The 30S ribosomal protein gene *rpsL* was used as an internal control. Error bars represent the standard deviation. ^**^P < 0.01; ^***^P < 0.001 by Student's *t*-test.

The transcriptome analysis results were presented as RNA expression levels relative to the PAO1 incubated in HBSS (Table 1, Table S3). Previously, it had been demonstrated that lethal concentrations of tobramycin highly activated the expression of genes involved in heat shock response, whereas bacteriostatic concentrations of tobramycin had minor effect (Kindrachuk et al., 2011). Consistent with the report, heat shock response genes *ibpA, groES, grpE, hslV*, and *hslU* were all up regulated after treatment with 2 μg/ml of tobramycin for 20 min in HBSS, however, 0.3 μg/ml of tobramycin did not significantly affect the expression of these genes (Table 1). Interestingly, treatment with 0.3 μg/ml of tobramycin in the presence of A549 and dHL60 cells induced the expression of those heat shock genes even higher than treatment by 2 μg/ml of tobramycin in HBSS (Table 1). Real time PCR analysis confirmed the mRNA levels of *ibpA* and *groES* (Figure 4). These results suggest that contact with the host cells intensified bacterial response to tobramycin, which is consistent with our bacterial survival results.

### Contact with dHL60 decreased bacterial membrane potential

**Figure 5:**
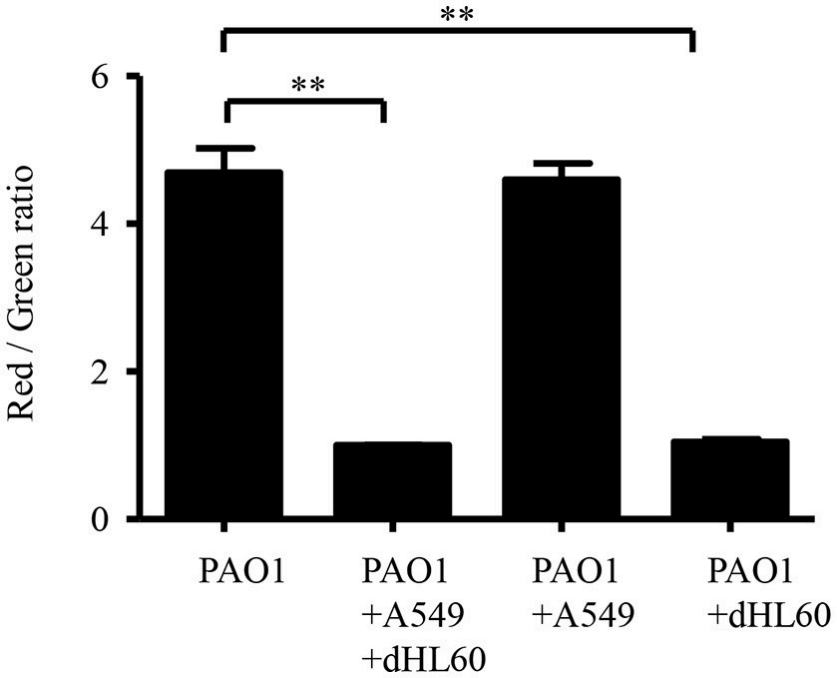
**Interaction with host cells affects P. aeruginosa membrane potential**. Membrane potential was determined by staining with DiOC2(3). Samples were prepared as follows: PAO1; PAO1 incubated with A549 cells for 50 min; PAO1 incubated with dHL60 for 40 min; PAO1 incubated with A549 cells for 10 min, followed by addition of dHL60 cells for 40 min. Then cells were stained with DiOC2(3) for 20 min in dark. The fluorescence was determined by flow cytometry. Membrane potential was represented by the ratio between green and red fluorescence. Error bars represent the standard deviation. ^**^P < 0.01 by Student's *t*-test.

We hypothesized that the increased bacterial susceptibility to tobramycin might be due to decreased membrane potential (Taber et al., 1987). Indeed, incubation with A549 and dHL60 decreased bacterial membrane potential (Figure 5). To dissect the role of each type of cell in the decreased membrane potential, we incubated the bacteria with individual cell line. Incubation with dHL60 but not A549 cells decreased membrane potential (Figure 5). These results suggest that dHL60 played a major role in the decreased membrane potential, which might contribute to increased bacterial susceptibility to tobramycin.

### Lipopolysaccharide phosphorylation contributes to increased bacterial susceptibility to tobramycin

**Figure 6:**
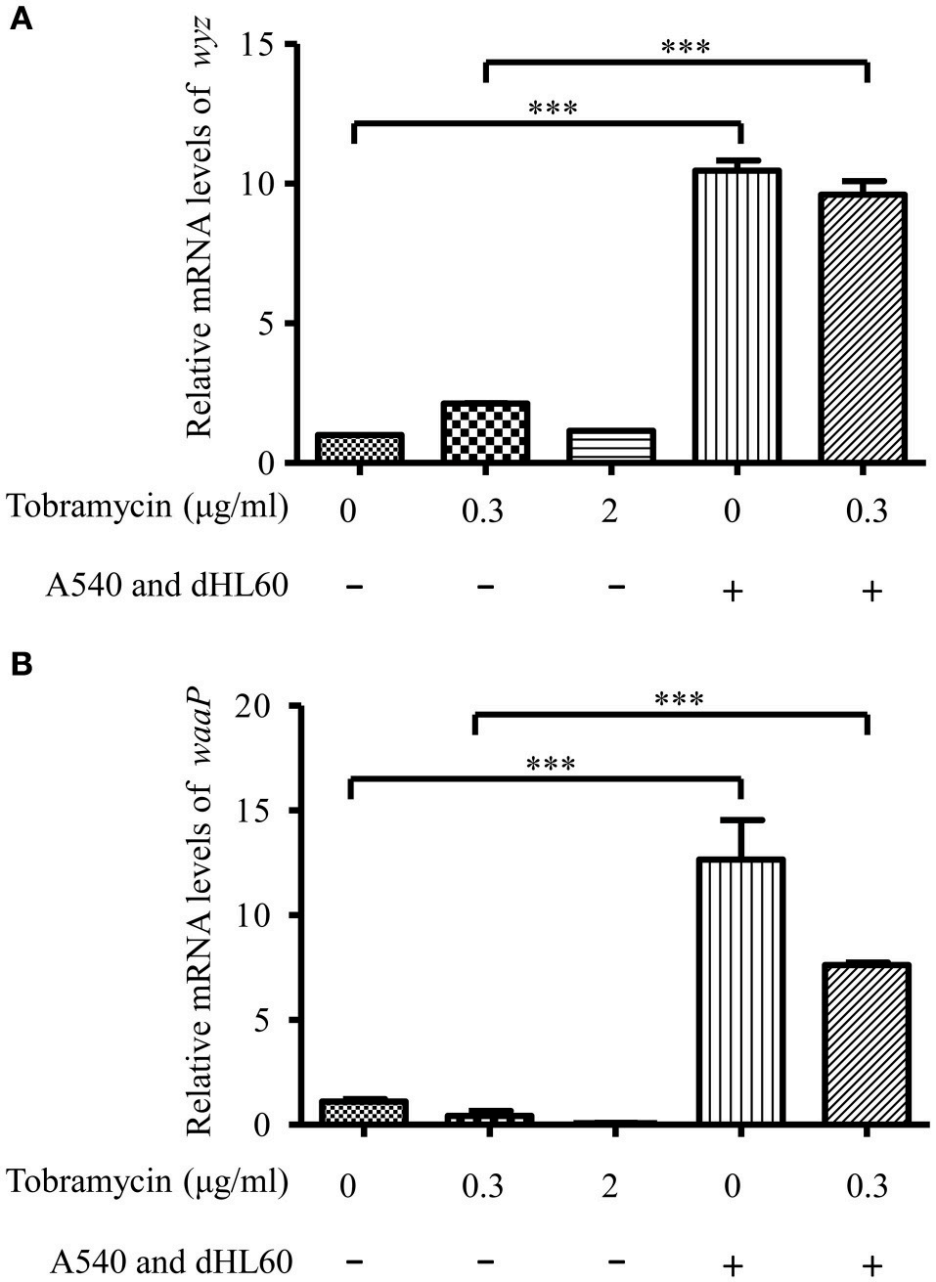
**mRNA level of waaP and wyz during *in vitro* cell infection**. RNA samples were prepared as in Figure 3. The mRNA levels of wyz **(A)** and waaP **(B)** were determined by real-time PCR. The 30S ribosomal protein gene rpsL was used as an internal control. Error bars represent the standard deviation. ^***^P < 0.001 by Student's *t*-test.

**Figure 7:**
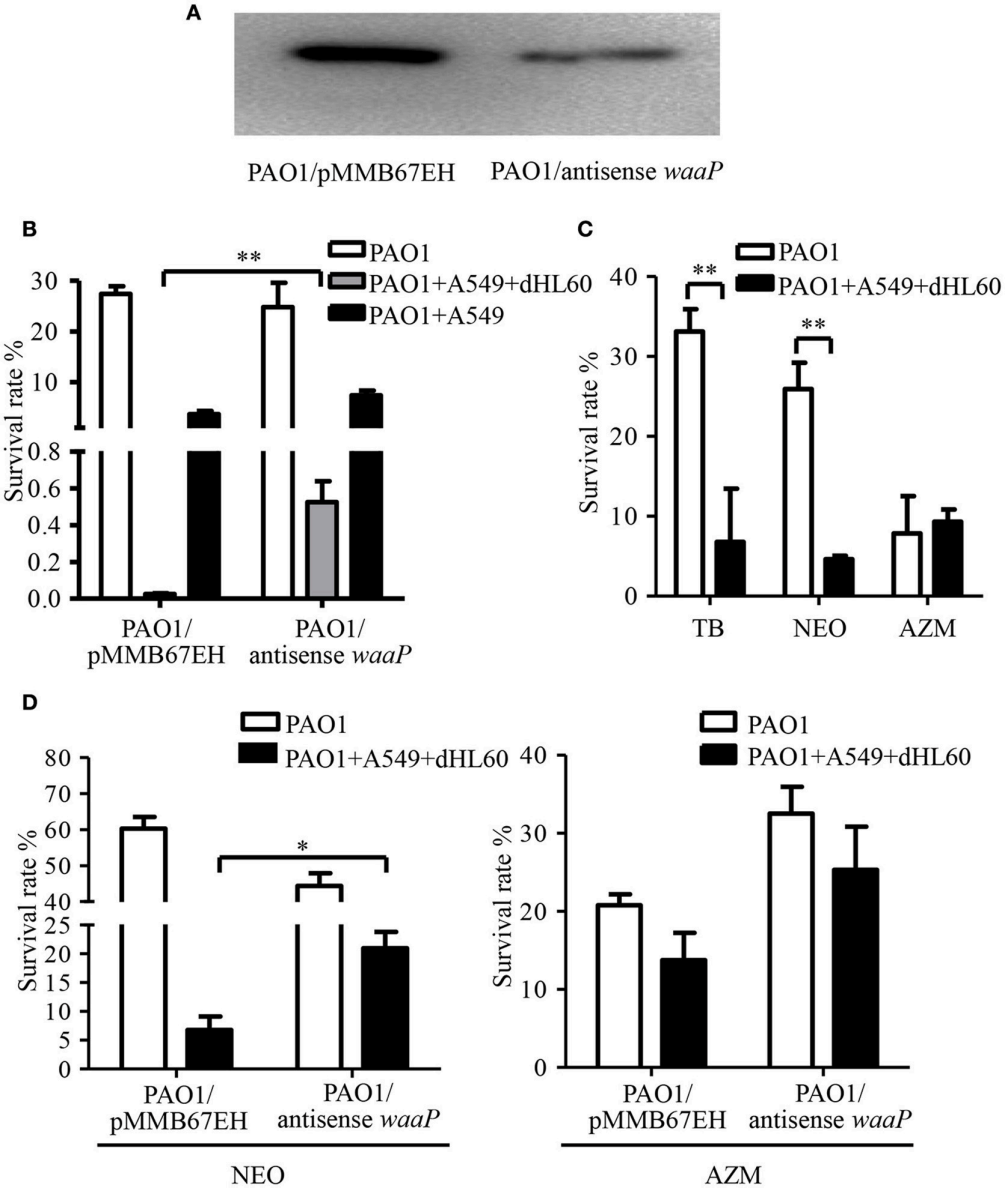
**Role of the LPS kinase WaaP in the increased bacterial susceptibility to tobramycin. (A)** Reduction of WaaP expression by an antisense RNA. After overnight culturing, PAO1 containing pMMB67EH-waaP (antisense RNA) and pRKaraRed-waaP-His or pMMB67EH and pRKaraRed-waaP-His were subcultured (1:50) in LB in the presence of indicated concentrations of IPTG and arabinose, and cultured for 3h at 37°C. The WaaP levels were determined by Western blotting with an anti-His antibody. **(B)** Survival rates of PAO1overexpressing antisense RNA against waaP and PAO1 containing the empty vector pMMB67EH. 1 × 107 of bacteria were incubated with 4 × 104 A549 for 10 min, followed by addition of 1 × 106 differentiated HL60 (dHL60) cells. After 10 min, 2 μg/ml tobramycin was added and incubated for another 50 min. The live bacteria number was determined by serial dilution and plating. The bacterial survival rate was calculated by dividing the number of live bacteria in the presence of tobramycin by that in the absence of tobramycin. **(C)** Survival rates of PAO1 alone or incubated with indicated cells. Bacteria were treated with 2 μg/ml tobramycin, 100 μg/ml neomycin or 300 μg/ml azithromycin. Same number of bacteria were used in the survival assay with our without the host cells. **(D)** Survival rates of PAO1 overexpressing antisense RNA against waaP and PAO1 containing the empty vector pMMB67EH. 1 × 107 of bacteria were incubated with 4 × 104 A549 for 10 min, followed by addition of 1 × 106 differentiated HL60 (dHL60) cells. After 10 min, 100 μg/ml neomycin or 300 μg/ml azithromycin was added and incubated for another 50 min. The bacterial survival rate was calculated by dividing the number of live bacteria in the presence of antibiotics by that in the absence of tobramycin. Error bars represent the standard deviation. ^*^P < 0.05; ^**^P < 0.01 by Student's *t*-test.

Besides membrane potential, the cellular entry of positively charged aminoglycoside antibiotic depends on its interaction with negatively charged LPS (Schindler and Teuber, 1975; Moore et al., 1986; Schnaitman and Klena, 1993). In our transcriptome analysis results, we found LPS synthesis genes as well as the LPS core kinase gene *waaP* were up regulated in response to the dHL60 and A549 cells (Table 1). Real time PCR assay confirmed the results (Figures 6A,B). We thus hypothesized that the up regulation of the LPS core kinase WaaP might increase the phosphorylation of LPS, which facilitates the entrance of tobramycin. Since *waaP* is an essential gene in *P. aeruginosa* (Walsh et al., 2000; Zhao et al., 2002; Delucia et al., 2011), we overexpressed an antisense RNA to reduce its expression level (Figure 7A). Indeed, reduction of WaaP level reduced bacterial susceptibility to tobramycin in the presence of dHL60 and A549 cells (Figure 7B).

If up regulation of WaaP contributes to the increased bacterial susceptibility to tobramycin, the bacterial should be susceptible to other aminoglycoside but not a different type of antibiotics. Indeed, incubation with dHL60 and A549 cells increased bacterial susceptibility to neomycin, but not the macrolide antibiotic azithromycin (Figure 7C). Reduction of WaaP level also reduced bacterial susceptibility to neomycin but did not affect the susceptibility to azithromycin in the presence of dHL60 and A549 cells (Figure 7D).

### Antibiotic susceptibility of bacteria isolated from BALF

**Figure 8:**
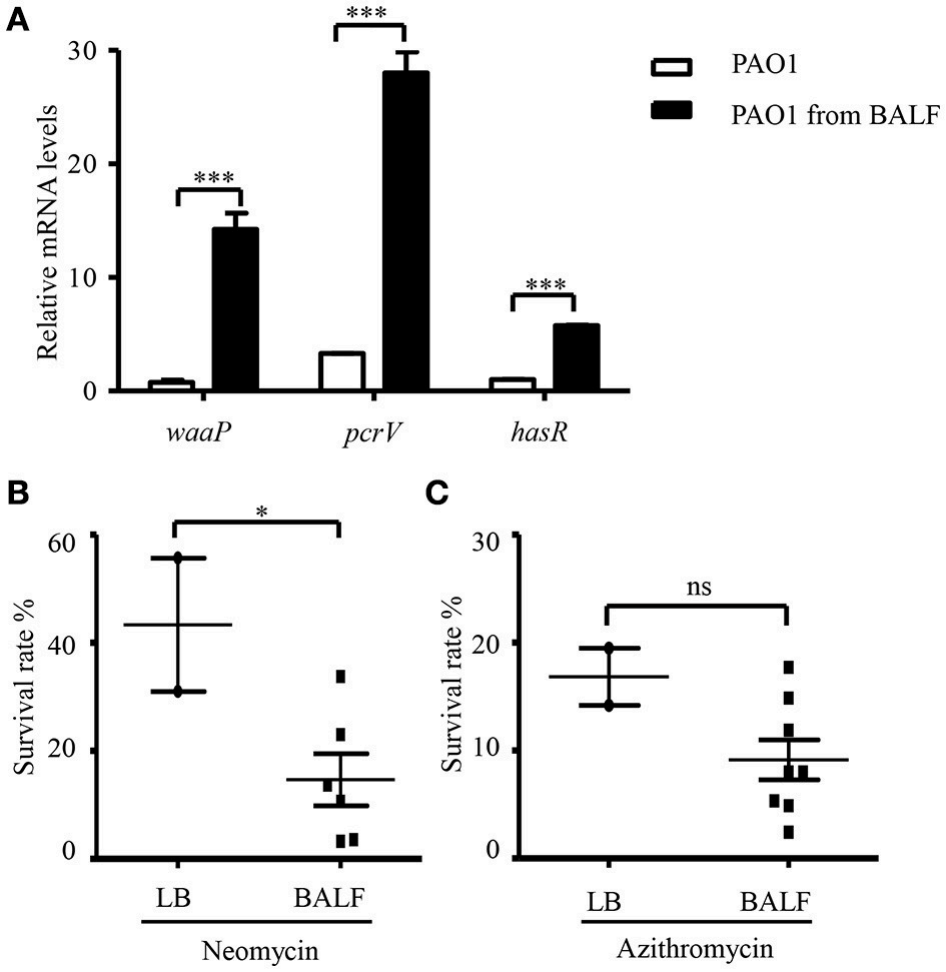
**Antibiotic susceptibility of bacteria isolated from BALF. (A)** The mRNA levels of waaP, pcrV, and hasR. RNA was isolated from PAO1 cultured in LB or collected from BALF 6 h post infection. The mRNA levles was determined by real time PCR. The 30S ribosomal protein gene rpsL was used as an internal control. ^***^P < 0.001 by Student's *t*-test. **(B,C)** Survival rate of PAO1 grown in LB or isolated from BALF. Mice were infected with 4 × 107 PAO1 intranasally. Six hours post infection, bacteria were isolated from BALF, resuspended in HBSS and treated with 100 μg/ml neomycin **(B)** or 300 μg/ml azithromycin **(C)** for 50 min. Bacteria grown in LB were resuspended in HBSS and subjected to antibiotic treatments as described for the bacteria isolated from BALF. Live bacteria number was determined by serial dilution and plating. Similar numbers of bacteria from BALFs and LB cultures were used in the assay. The initial CFU of each sample was shown in Table S2. Error bars represent the standard deviation. ^*^P < 0.05; ns, not significant, by Student's *t*-test.

The *in vivo* environment is much more complicated than our *in vitro* cell infection condition. In addition to epithelial and phagocytic cells, bacteria encounter a variety of molecules, such as mucin and defensins as well as iron limiting environment. In order to investigate whether the host *in vivo* environment affects the expression of *waaP*, we isolated bacteria from BALF 6 h post infection. mRNA levels of the T3SS gene *pcrV* and iron acquisition gene *hasR*, which are known to be activated during acute pneumonia (Shaver and Hauser, 2004; Damron et al., 2016) were higher in the bacteria isolated from BALF (Figure 8A), suggesting an adaptation to host environment. Consistent with our *in vitro* cell infection results, the expression of *waaP* was induced during infection (Figure 8A). And the isolated bacteria showed higher susceptibility to neomycin, but not to azithromycin (Figures 8B,C). Overall, these results suggest that host environment could affect bacterial susceptibility to aminoglycoside antibiotics.

## Discussion

Host environment imposes various stresses on invading bacteria, such as bactericidal peptides, complement, minimal free iron, attack from phagocytes, etc. To survive and replicate, bacteria produce multiple virulence factors and adjust metabolism. Upon invasion of host tissues, *P. aeruginosa* assembles the T3SS on the surface, through which effector proteins are directly injected into and kill host cells, preferentially phagocytes (Mougous et al., 2006; Diaz and Hauser, 2010). Various iron chelating molecules and cognate transportation systems are up regulated for the acquisition of iron from host. Besides, expression of multiple protein secretion systems and porin proteins are orchestrated and contribute to bacterial pathogenesis (Bleves et al., 2010; Filloux, 2011). Thus, the membrane permeability and gene expression profile are significantly altered by host environment.

Membrane permeability is directly related to bacterial antibiotic resistance (Delcour, 2008). As certain antibiotics cross outer membrane through specific porins, these proteins are related to antibiotic resistance. For example, carbapenem antibiotics enter periplasm through OprD. Mutation in the *oprD* gene increases bacterial resistance to carbapenem antibiotics (Fukuoka et al., 1993). Interestingly, loss of OprD increases bacterial fitness during infection (Skurnik et al., 2013). The chromosomally encoded β-lactamase (AmpC) plays an important role in bacterial resistance to β-lactam antibiotics. Expression of AmpC is regulated by AmpR in responding to peptidoglycan integrity stress. Besides, AmpR directly regulates genes or small RNAs involved in quorum sensing, iron acquisition, and oxidative stress response (Balasubramanian, 2014). These examples imply that the regulation and functions of virulence factors and antibiotic resistant determinants are interwoven together.

During infection, bacteria encounter antibiotic in the *in vivo* host environment. The bacterial response to antibiotics in this special milieu remains largely unknown. In this study, we examined bacterial susceptibility and response to tobramycin in the presence of epithelial A549 cells and neutrophil (dHL60) cells, which represent the major types of cells comes in contact with bacteria during infection (Driscoll et al., 2007). Tobramycin is one of the most frequently used aminoglycoside antibiotics in the clinic for treatment of *P. aeruginosa* infections (Gziut et al., 2013). Aminoglycoside antibiotics bind to negatively charged LPS by displacing Mg^2+^ and Ca^2+^ before penetrating bacteria outer membrane (Kotra et al., 2001). Here we found that presence of A549 and dHL60 cells sensitized *P. aeruginosa* to tobramycin. One of the causes might be decreased membrane potential as revealed by flow cytometric analysis (Novo et al., 1999). As decreased membrane potential indicates increased membrane permeability (Novo et al., 2000), it is likely that the presence of host cells lead to higher intracellular antibiotic concentration. Consistently, in the presence of the host cells, genes known to response to tobramycin were up regulated by a low concentration of tobramycin (0.3 μg/ml), which alone could not induce the expression of these genes.

We further found that ROS generated by neutrophil contributes to the decreased membrane potential. Previously, van der Heijden et al. demonstrated that *Salmonella* regulates membrane permeability in response to ROS (van der Heijden et al., 2016). *Salmonella* outer membrane proteins, OmpA and OmpC, are rapidly opened or closed in response to oxidative stresses (van der Heijden et al., 2016). We suspect that ROS might trigger gene expression alteration, leading to a different composition of membrane proteins, which affect membrane permeability. Another possibility is that ROS might impair *P. aeruginosa* membrane integrity through damaging phospholipids or membrane proteins.

Compared to dHL60 alone, preincubation with A549 further increased bacterial susceptibility to tobramycin (Figure 2C). It might be possible that the presence of A549 affects the behavior of dHL60, e.g., the level of degranulation or formation of neutrophil extracellular traps. In addition, contact with both A549 and dHL60 cells might further alter bacterial gene expression compared to dHL60 alone, and thus affects bacterial susceptibility to antibiotics.

To examine the global gene expression in the presence of host cells and tobramycin, we performed transcriptome analysis. No major multidrug efflux system was down regulated upon interaction with A549 and dHL60 cells (Table S3). Interestingly, we observed that the kinase WaaP which phosphorylates the LPS inner core were up regulated after interaction with the cells. The anionic phosphate group is a major source of negative charge on LPS (Wang and Quinn, 2011; Gyurova et al., 2013). Aminoglycoside antibiotics are cationic antibiotics. The initial step of these antibiotics to enter bacterial cell is to bind to LPS (Rivera et al., 1986). Thus, increased phosphorylation of LPS might promote binding between aminoglycoside antibiotics and LPS, which facilitates the entry of the antibiotics. Indeed, knock-down of WaaP by an antisense RNA partially reduced the bacterial susceptibility in the presence of host cells. Further studies are warranted to elucidate the regulatory mechanism of WaaP as well as its function in bacterial pathogenesis.

Bacteria isolated from BALF in an acute pneumonia model were more susceptible to tobramycin than *in vitro* cultured bacteria. Our results indicated that WaaP was up regulated during infection, which might contribute to the increased susceptibility. These results suggest that host *in vivo* environment might affect bacterial resistance to antibiotics. However, in the treatment of chronic *P. aeruginosa* infection, the killing effect might be complicated by the formation of biofilm. We are making efforts to study the bacterial susceptibility in biofilm in the presence of host cells.

## Author contributions

Conceived and designed the experiments: WW, XP, SJ. Performed the experiments: XP, YD, ZF, CL, YJ, BX, JS. Analyzed the data: XP, WW, SJ, ZC, FB, YJ. Wrote the paper: XP, WW, SJ.

## Funding

This work was supported by National Science Foundation of China (31670130, 31370168, 31370167 and 31600110); Program of international S&T cooperation (2015DFG32500) and Science and Technology Committee of Tianjin (15JCYBJC53900 and 15JCZDJC33000). The funders had no role in study design, data collection and interpretation, or the decision to submit the work for publication.

### Conflict of interest statement

The authors declare that the research was conducted in the absence of any commercial or financial relationships that could be construed as a potential conflict of interest.

## Supplementary material

Figure S1 **Effect of host cells on bacterial survival**. PAO1 cells (1 × 10^7^) were incubated with A549 (4 × 10^4^) for 10 min, followed by addition of differentiated HL60 (dHL60) cells (1 × 10^6^). After 60 min, the number of live bacteria was determined by serial dilution and plating. Error bars represent the standard deviation. ^***^*P* < 0.001 as determined by Student's *t*-test.

Figure S2 **H_2_O_2_ increases bacterial susceptibility to tobramycin**. Survive rate of PAO1 treated with or without 2 μg/ml tobramycin in the presence of indicated concentrations of H_2_O_2_ for 50 min. Error bars represent the standard deviation. ^**^*P* < 0.01 by Student's *t*-test.

Figure S3 **Susceptibility of PAO1 to tobramycin. (A)** Survival curve of PAO1 treated with tobramycin at indicated time points. Bacterial cells (1 × 10^7^) were treated with 2 μg/ml of tobramycin. At indicated time points, the bacterial survival rates were determined by serial dilution and plating. **(B)** PAO1 was treated with tobramycin at indicated concentrations for 20 min. Alternatively, PAO1 was incubated with A549 (4 × 10^4^) for 10 min, followed by addition of dHL60 cells (1 × 10^6^). After 20 min, tobramycin at the indicated concentrations was added to the mixture and incubated for 20 min. The number of live bacteria was determined by serial dilution and plating. Error bars represent the standard deviation.

Table S1 **Bacterial strains, plasmids and primers used in this study**.

Table S2 **The initial bacterial CFU used in antibiotic treatment assay from individual BALF or LB culture**.

Table S3 **Transcriptome analysis: differentially regulated genes**.
