# Supplementary figures and images for: In vivo Host Environment Alters Pseudomonas aeruginosa Susceptibility to Aminoglycoside Antibiotics
Source: Front Cell Infect Microbiol. 2017 Mar 14;7:83. doi: 10.3389/fcimb.2017.00083 (PMC5348532; doi:10.3389/fcimb.2017.00083)

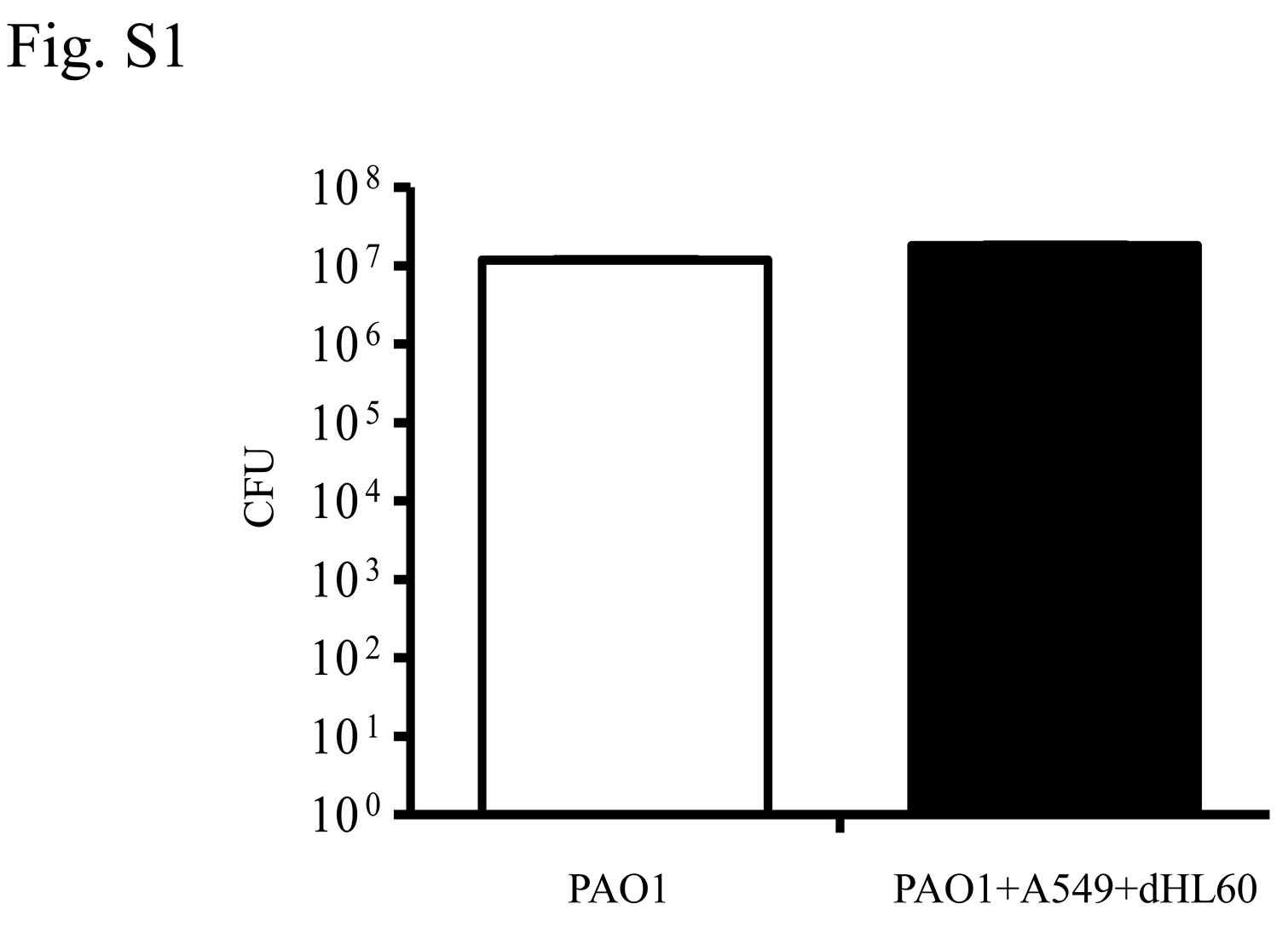

Supplement: Figure S1 — Effect of host cells on bacterial survival. PAO1 cells (1 × 10^7^) were incubated with A549 (4 × 10^4^) for 10 min, followed by addition of differentiated HL60 (dHL60) cells (1 × 10^6^). After 60 min, the number of live bacteria was determined by serial dilution and plating. Error bars represent the standard deviation. ***P < 0.001 as determined by Student's t-test. [file Image1.TIF]

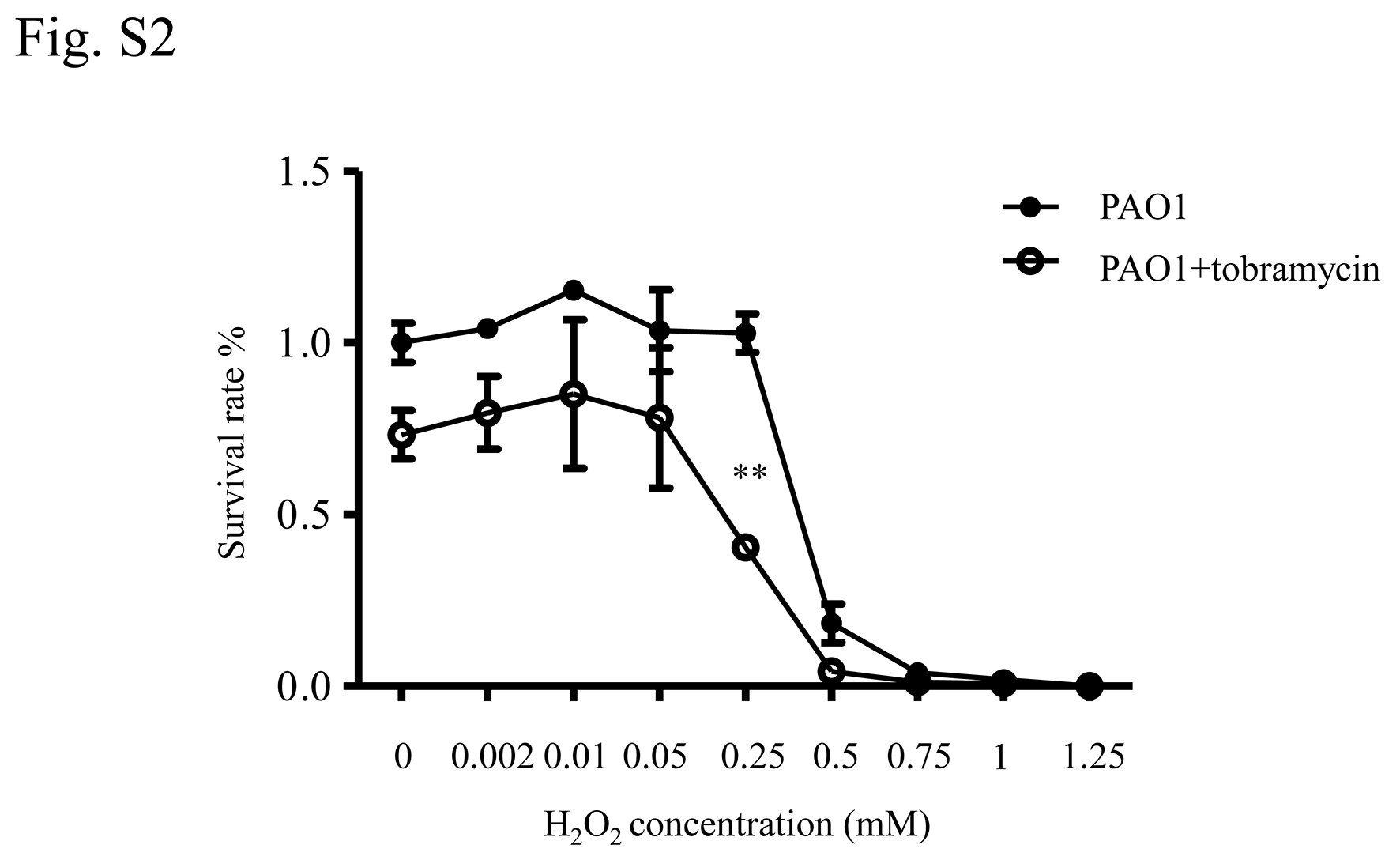

Supplement: Figure S2 — H2O2 increases bacterial susceptibility to tobramycin. Survive rate of PAO1 treated with or without 2 μg/ml tobramycin in the presence of indicated concentrations of H2O2 for 50 min. Error bars represent the standard deviation. **P < 0.01 by Student's t-test. [file Image2.TIF]

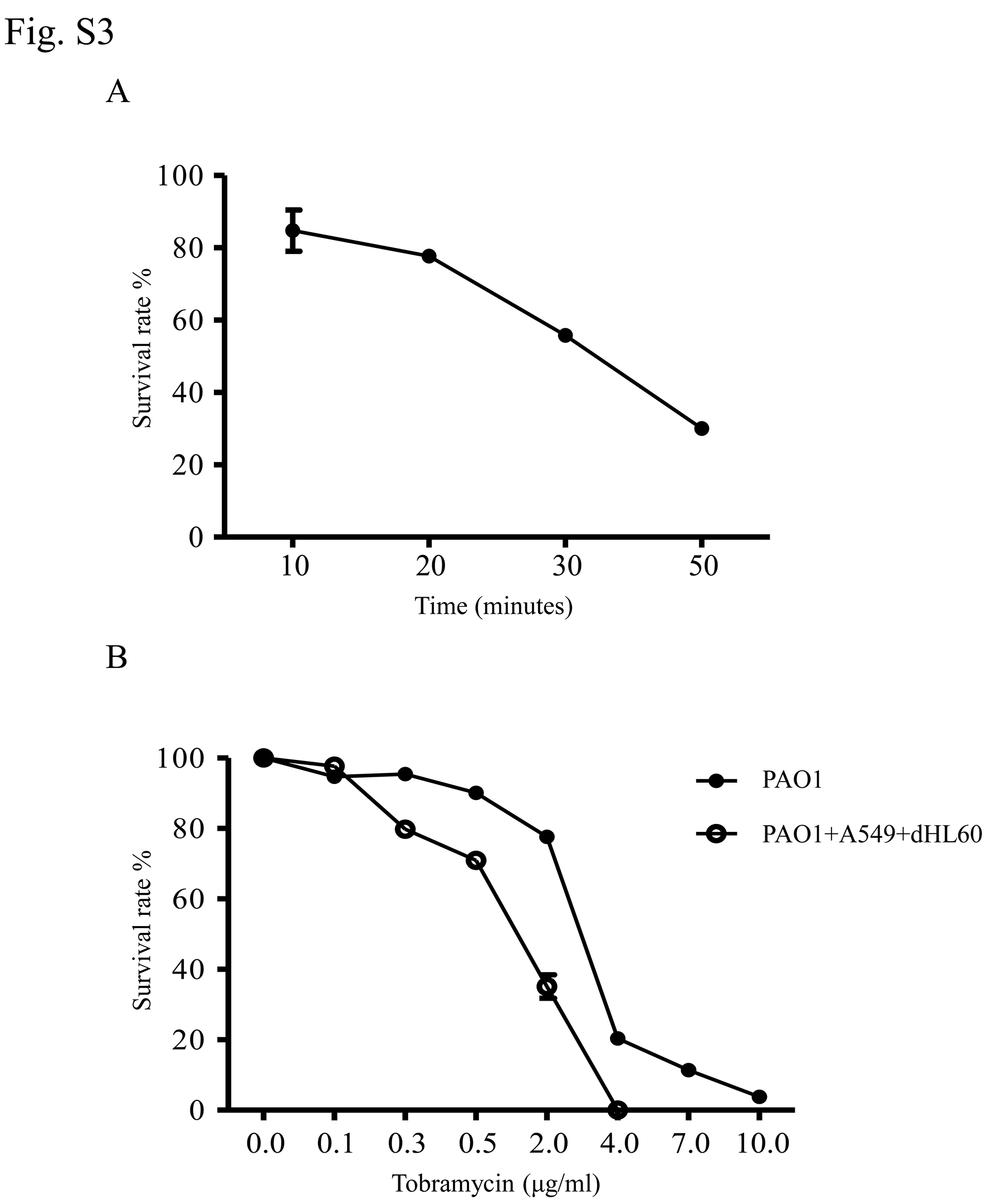

Supplement: Figure S3 — Susceptibility of PAO1 to tobramycin. (A) Survival curve of PAO1 treated with tobramycin at indicated time points. Bacterial cells (1 × 10^7^) were treated with 2 μg/ml of tobramycin. At indicated time points, the bacterial survival rates were determined by serial dilution and plating. (B) PAO1 was treated with tobramycin at indicated concentrations for 20 min. Alternatively, PAO1 was incubated with A549 (4 × 10^4^) for 10 min, followed by addition of dHL60 cells (1 × 10^6^). After 20 min, tobramycin at the indicated concentrations was added to the mixture and incubated for 20 min. The number of live bacteria was determined by serial dilution and plating. Error bars represent the standard deviation. [file Image3.TIF]
