# Supplementary material for: In vivo Host Environment Alters Pseudomonas aeruginosa Susceptibility to Aminoglycoside Antibiotics
Source: Front Cell Infect Microbiol. 2017 Mar 14;7:83. doi: 10.3389/fcimb.2017.00083 (PMC5348532; doi:10.3389/fcimb.2017.00083)
Supplement: Table S1 — Bacterial strains, plasmids and primers used in this study. [file Table1.DOC]

Table S1. Bacterial strains, plasmids and primers used in this study.

| **Strain/ Plasmid /Primer** | **Description** | **Source (Reference)** |
| --- | --- | --- |
| ***P. aeruginosa*** | | |
| PAO1 | Wild type strain of *Pseudomonas aeruginosa* |  |
| **Plasmid** |  |  |
| pMMB67EH | Expression vector with *tac* promoter; Ap^r^ |  |
| pRKaraRed | Expression vector with P_BAD_ promoter; Ap^r^ |  |
| pMMB67EH-*waaP* antisense RNA | Antisense RNA driven by *tac* promoter on pMMB67EH; Ap^r^ | This study |
| pRKaraRed-*waaP*-His | WaaP with his tag driven by P_BAD_ promoter on pRKaraRed; Ap^r^ | This study |
| **Primer** | **Sequence (5’→3’)** | **Function** |
| Nco I-anti waaP-F | CATGCCATTGCTCCACCGCCTCGAACG | Antisense RNA cloning |
| Xho I-anti waaP-R | CCGCTCGAGAGCATGCCGCAGCGCG | Antisense RNA cloning |
| Sal I-WaaP His-F | GCGTCGACACCTGATCCTCGGGGAG | WaaP-His cloning |
| Xba I-WaaP His-R | GCTCTAGATCAATGATGATGATGATGATGTCCGTAACGCTGCTTGC | WaaP-His cloning |
| waaP-F | CCGTGGCATCGGCTGG | RT-PCR |
| waaP-R | CGGTGACGATGAAGGAATGCT | RT-PCR |
| ibpA-F | GTTTCTGATGGAGTTGGAT | RT-PCR |
| ibpA-R | ATATCGTTCACTGTCGGA | RT-PCR |
| groES-F | GAACATCAGCTTCGTCAA | RT-PCR |
| groES-R | TCGGCAGCATTATTGATT | RT-PCR |
| wyz-F | AGCGACAGTGTGGGTGAATAATA | RT-PCR |
| wyz-R | CAAGTACGGATACAAGCCGAAGT | RT-PCR |
| hasR-F | CAGAACACCTCGACCTTC | RT-PCR |
| hasR-R | TTGTCGTAGAAGAACTCCAG | RT-PCR |
| pcrV-F | CACGCTCTATGGCTATGC | RT-PCR |
| pcrV-R | AAGGTATCCAGATTGCTCAG | RT-PCR |
|  |  |  |
|  |  |  |
|  |  |  |
|  |  |  |

**References**

Fürste, J.P., Pansegrau, W., Frank, R., Blöcker, H., Scholz, P., Bagdasarian, M., et al. (1986). Molecular cloning of the plasmid RP4 primase region in a multi-host-range tacP expression vector. *Gene* 48(1)**,** 119-131. doi: 10.1016/0378-1119(86)90358-6.

Liang, R., and Liu, J. (2010). Scarless and sequential gene modification in Pseudomonas using PCR product flanked by short homology regions. *BMC Microbiology* 10(1)**,** 209.

Liberati, N.T., Urbach, J.M., Miyata, S., Lee, D.G., Drenkard, E., Wu, G., et al. (2006). An ordered, nonredundant library of Pseudomonas aeruginosa strain PA14 transposon insertion mutants. *Proceedings of the National Academy of Sciences of the United States of America* 103(8)**,** 2833-2838.
