# Supplementary material for: In vivo Host Environment Alters Pseudomonas aeruginosa Susceptibility to Aminoglycoside Antibiotics
Source: Front Cell Infect Microbiol. 2017 Mar 14;7:83. doi: 10.3389/fcimb.2017.00083 (PMC5348532; doi:10.3389/fcimb.2017.00083)
Supplement: Table S2 — The initial bacterial CFU used in antibiotic treatment assay from individual BALF or LB culture. [file Table2.DOC]

**Table S2. The initial bacterial CFU used in antibiotic treatment assay from individual BALF or LB culture**.

| **Antibiotics** | **Initial CFU in BALF of each mouse (×10^6^)** | **Initial CFU in each LB culture (×10^6^)** |
| --- | --- | --- |
| **Tobramycin treated samples** | | |
|  | Mouse #1: 10.42  Mouse #2: 4.17  Mouse #3: 0.83  Mouse #4: 2.08  Mouse #5: 5.63  Mouse #6: 3.75  Mouse #7: 2.71  Mouse #8: 18.76 | Sample #1: 2.71  Sample #2: 2.50 |
| **Neomycin or azithromycin treated samples** | | |
|  | Mouse #1: 1.71  Mouse #2: 1.33  Mouse #3: 0.13  Mouse #4: 1.25  Mouse #5: 0.17  Mouse #6: 0.96 | Sample #1: 3.23  Sample #2: 3.25 |
